# N*-*linked glycosylation of N48 is required for equilibrative nucleoside transporter 1 (ENT1) function

**DOI:** 10.1042/BSR20160063

**Published:** 2016-08-31

**Authors:** Alex Bicket, Imogen R. Coe

**Affiliations:** * Department of Biology, York University, Toronto, Canada M3J 1P3; † Department of Chemistry and Biology, Ryerson University, Toronto, Canada M5B 2K3

**Keywords:** equilibrative nucleoside transporter 1, function, immunofluorescence, N-linked glycosylation, oligomerization, trafficking

## Abstract

Human equilibrative nucleoside transporter 1 (hENT1) transports nucleosides and nucleoside analogue drugs across cellular membranes and is necessary for the uptake of many anti-cancer, anti-parasitic and anti-viral drugs. Previous work, and *in silico* prediction, suggest that hENT1 is glycosylated at Asn^48^ in the first extracellular loop of the protein and that glycosylation plays a role in correct localization and function of hENT1. Site-directed mutagenesis of wild-type (wt) hENT1 removed potential glycosylation sites. Constructs (wt 3xFLAG-hENT1, N48Q-3xFLAG-hENT1 or N288Q-3xFLAG-hENT2) were transiently transfected into HEK293 cells and cell lysates were treated with or without peptide–*N*-glycosidase F (PNGase-F), followed by immunoblotting analysis. Substitution of N48 prevents hENT1 glycosylation, confirming a single N-linked glycosylation site. N48Q-hENT1 protein is found at the plasma membrane in HEK293 cells but at lower levels compared with wt hENT1 based on *S*-(4-nitrobenzyl)-6-thioinosine (NBTI) binding analysis (wt 3xFLAG-ENT1 *B*_max_, 41.5±2.9 pmol/mg protein; N48Q-3xFLAG-ENT1 *B*_max_, 13.5±0.45 pmol/mg protein) and immunofluorescence microscopy. Although present at the membrane, chloroadenosine transport assays suggest that N48Q-hENT1 is non-functional (wt 3xFLAG-ENT1, 170.80±44.01 pmol/mg protein; N48Q-3xFLAG-ENT1, 57.91±17.06 pmol/mg protein; mock-transfected 74.31±19.65 pmol/mg protein). Co-immunoprecipitation analyses suggest that N48Q ENT1 is unable to interact with self or with wt hENT1. Based on these data we propose that glycosylation at N48 is critical for the localization, function and oligomerization of hENT1.

## Abstract (precis)

Our study confirmed that Asn^48^ of hENT1 is the only N-glycosylated residue when expressed in HEK293 cells, and loss of the N-glycan resulted in less hENT1 at the plasma membrane, as well as a loss of function and protein–protein self-interaction.

## INTRODUCTION

Membrane transporter proteins enable movement of molecules across biological membranes. Nucleosides are hydrophilic molecules involved in cell signalling, DNA synthesis and energy metabolism, and require trans-membrane transport. The equilibrative nucleoside transporters (ENTs) comprise the solute carrier (SLC), SLC29, family [1]. ENTs passively facilitate movement of nucleosides down their concentration gradients [2] whereas CNTs (SLC28) are cation/nucleoside co-transporters which do not possess any sequence or known structural homology to ENTs [3].

ENTs are critical for the uptake of many classes of nucleoside derivative drugs. ENT1 and ENT2 are clinically important drug transporters that are critical for drug delivery, and therefore efficacy, of many anti-cancer, anti-parasitic and anti-viral agents [4]. Moreover, ENTs modulate adenosine flux and thereby regulating purinergic responses [5,6].

Like most SLC proteins [7], human ENT1, hENT1, is reported as being glycosylated at the large extracellular loop [8]. Glycosylation mutants of hENT2 expressed in mammalian cells show reduced transport and protein levels at the membrane [9]. In contrast, glycosylation mutants of hENT1, expressed in *Saccharomyces cerevisiae*, show increased expression at the plasma membrane and functional transport [10]. Therefore, the role of glycosylation of hENT1 in human cells is unclear.

N-linked glycosylation of membrane transporters is important in function [11,12], trafficking [13,14], stability [15,16] and sorting [17]. Therefore we hypothesized that non-glycosylated hENT1 would exhibit reduced recruitment to the plasma membrane resulting in lower hENT1-dependent transport. Reduced hENT1-dependent uptake of nucleoside analogue drugs used in disease treatment has significant clinical implications since drug efficacy is correlated with hENT1 presence [18]. Since cancerous cells can exhibit global changes in cellular glycosylation [19], understanding the role of ENT1 glycosylation is clinically relevant. In the present study, we show that N-linked glycosylation of hENT1 is necessary for function.

## MATERIALS AND METHODS

### *In silico* detection of putative glycosylation sites

We used NetNGlyc 1.0 to determine putative N-linked glycosylation sites in full length human ENT1 sequence (accession number NP_001071645).

### Cell culture and transfection

HEK293 (human embryonic kidney cell line), commonly used in membrane protein glycosylation studies [20–22], were grown in Dulbecco's Modified Eagle Media (DMEM) supplemented with 10% (v/v) FBS in 10 cm^2^ plates [*S*-(4-nitrobenzyl)-6-thioinosine (NBTI) binding and Western blotting] or six-well plates (transport assays) at 37°C with 5% (v/v) CO_2_. Cells were transfected using the standard Polyjet protocol (SignaGen Laboratories) and incubated post transfection for ∼36 h. Equivalent transfection efficiency in wild type (wt) and mutant-transfected cells was confirmed by microscopy.

### Generating N48Q-hENT1 and N288Q-hENT1 mutant constructs

Full-length hENT1 conjugated with a 3xFLAG tag in a pCDNA 3.1 vector was used as the template and point mutations were introduced using overlap extension PCR [23]. To create the N48Q mutation, the AAT codon was substituted for a CAA, whereas the N288Q mutation used an AAT codon substituted for a CAG.

### Immuno-blotting analysis

To determine which residues were N-glycosylated, we overexpressed wt, N48Q or N288Q mutant hENT1 protein in HEK293 cells, and treated lysates with and without peptide–*N*-glycosidase F (PNGase-F), followed by immunoblotting analyses as previously described [24].

### NBTI binding assay

NBTI is a high affinity, tight-binding, non-transportable, ENT1-specific nucleoside analogue used which can be used to analyse the presence of ENT1 [25–27] as previously described [28]. [^3^H]-NBTI binding parameters (*K*_d_ and *B*_max_) were determined from non-linear regression analysis using GraphPad Prism (v. 5.04).

### [^3^H]-2-chloroadenosine transport assay

To determine the functionality of N48Q-hENT1, we conducted [^3^H]-2-chloroadenosine transport assays using HEK293 cells as previously described [29].

### Immunofluorescence and point scanning confocal microscopy

We used immunofluorescence microscopy to investigate localization of wt 3xFLAG-hENT1 and N48Q-hENT1. wt 3xFLAG-hENT1 and N48Q-3xFLAG-hENT1 vectors were transfected into HEK293 cells as described above. Cells grown on coverslips were prepared as previously described [30] followed by incubation with anti-FLAG primary and Alexa488 or Alexa594 fluorescent secondary antibody [1:500, in 1% (v/v) milk in tris-buffered saline and Tween 20 (TTBS), 45 min]. Slides were viewed using a Zeiss LSM 700 Inverted Confocal microscope with a Plan Apochromat 63× oil immersion objective lens (N.A.=1.40). Z-stacks (8–12 at ∼1 μm intervals) were collected. Zen Black (Zeiss) software was used for image acquisition and image processing.

### Co-immunoprecipitation of wild type and glycosylation mutant FLAG-ENT1 using HA-ENT1 bait

HEK293 cells co-transfected (as described above) with HA-ENT1 and 3xFLAG-vector (either wt ENT1, N48Q-ENT1 or hLa as a negative control) were lysed with Nonidet P-40 (octyl phenoxypolyethoxylethanol) (NP-40) buffer ∼36 h post-transfection. Lysate was homogenized with 1 ml syringe and 26 ***g*** needle then centrifuged at max speed (15 min) on a bench top centrifuge to pellet cellular debris and organelles. Protein concentration was determined by modified Lowry protein assay (Bio-Rad Laboratories). To best equilibrate the strength of transfected protein bands between the constructs when immunoblotting, columns were loaded with transfected cell lysate as follows: wt 3xFLAG-ENT1 (100 μg), N48Q-3xFLAG-ENT1 (1000 μg) and 3xFLAG-hLa (600 μg, RNA chaperone found primarily in the nucleus, but also in the cytoplasm, used as a negative control), each with 20 μl of anti-HA beads (Thermo Scientific). Protein was agitated overnight (approximately 18 h) at 4°C and washed three times with TTBS. Immuno-precipitated protein was recovered by boiling with 2× elution buffer (Thermo Scientific) and supplemented with 1 M dichlorodiphenyltrichloroethane (DDT; 2 μl). Protein from elution and flow-through was resolved by SDS/PAGE and subjected to immuno-blotting as described above. The entire elution fraction added to the corresponding lane in the gel, whereas flow through protein was loaded as follows: 1 μg wt, 10 μg N48Q, 1 μg hLa.

## RESULTS

### hENT1 possesses a single glycosylation site at Asparagine-48

**Figure 1:**
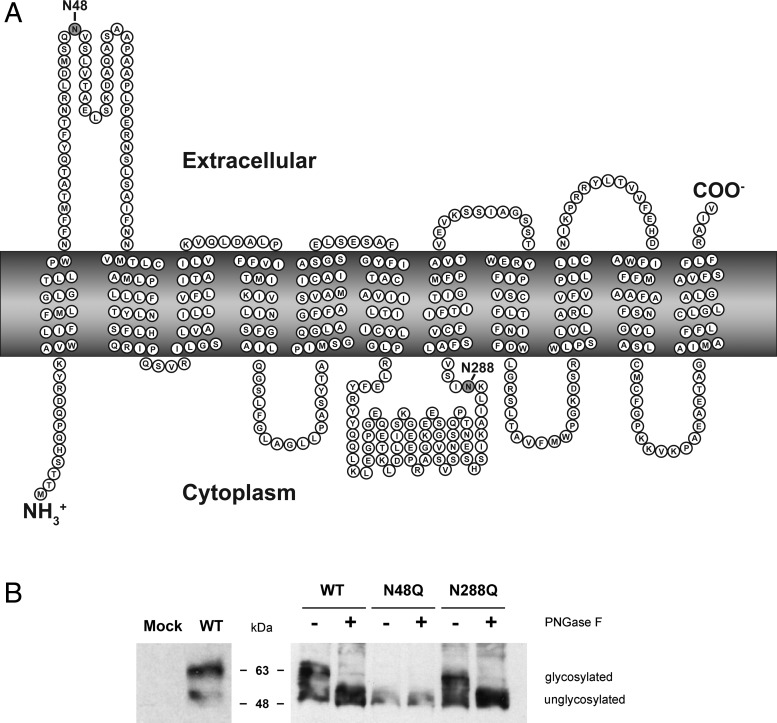
Predicted hENT1 topology and immunoblot identifying N48 as only N-linked glycan in human cells (**A**) Putative 2D membrane topology with labelled Asn residues identified as putative N-glycosylation sites based on NetNGlyc prediction. (**B**) Immunoblot with wt (3xFLAG-hENT1) and mutant (N48Q-3xFLAG-hENT1 and N288Q-3xFLAG-hENT1) cell lysates from transfected HEK293 cells, with and without PNGase-F treatment (right). Mock transfected and 3xFLAG-hENT1 transfected cells (left) confirmed antibody specificity. Whole cell lysates were fractioned with SDS/12% (v/v) PAGE and immunoblotted with anti-FLAG antibody.

*In silico* analyses suggested that N48 and N288 had the highest probability of glycosylation. N48 is the most plausible target since it is within the large extracellular loop, whereas N288 is a less likely target since it is near a transmembrane domain (TMD) and is likely to exist within the cytosol [31]. Although N288 is less likely, a definitive 3D structure of ENT1 has not been established so it is possible that this residue is exposed to the extracellular space, thus, to be thorough, both targets were tested (Figure 1A). Previous work suggested ENT1 was glycosylated at N48 when expressed in *S. cerevisiae* [10]. Our results suggest that wt hENT1, expressed in HEK293 cells, is a protein of 50–65 kDa and following PNGase-F treatment, the size of the protein is reduced to 50–55 kDa (Figure 1B). In contrast, N48Q hENT1 mutant protein is 50–55 kDa in the presence and absence of PNGase-F confirming hENT1 is exclusively N-glycosylated at N48 in human cells with no evidence of glycosylation at N288.

### N-linked glycosylation of N48 is required for hENT1 movement to the plasma membrane

**Figure 2:**
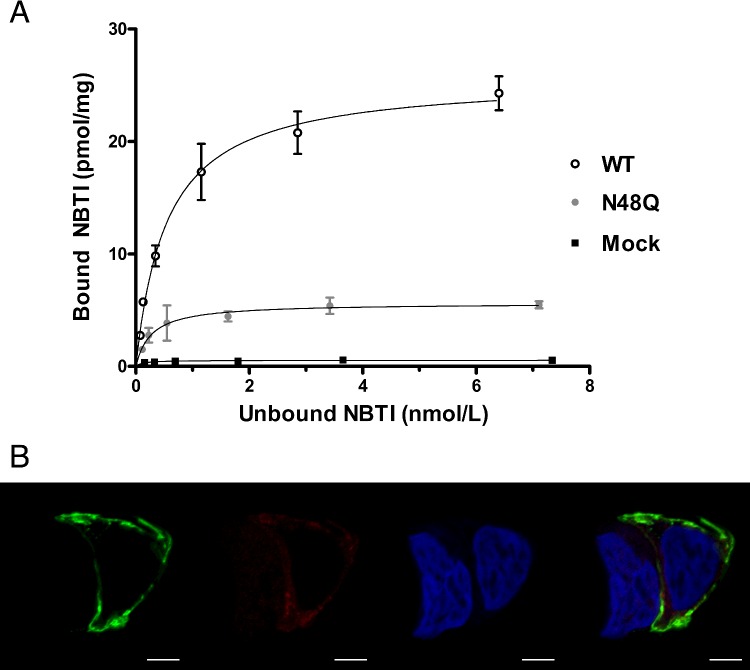
Loss of N-glycosylation reduces hENT1 presence at the plasma membrane (**A**) HEK293 cells transfected with N48Q-3xFLAG-hENT1 exhibited a 3-fold decrease in hENT1 NBTI binding sites compared with transfected 3xFLAG-hENT1. Both N48Q hENT1 and wt hENT1 transfected cells showed an increase in NBTI binding sites compared with mock transfected. Error bars represent the mean±S.D. Representative graph from three experiments, with each point conducted in duplicate. (**B**) Confocal microscopy of fixed HEK293 cells transfected with HA-hENT1 and N-linked glycosylation mutant N48Q-3xFLAG-hENT1. Cells were fixed and probed with anti-FLAG primary antibody then Alexa594 secondary and anti-HA primary antibody then Alexa488 secondary antibody, nuclei were stained with DAPI as described in the Materials and Methods. Red fluorescence represents N48Q mutant hENT1, green fluorescence represents wt hENT1 and blue fluorescence represents DNA. Red, green and blue fluorescence were achieved by excitation with 555 nm, 488 nm and 405 nm respectively, with each signal acquired separately. Altered distribution is observed between HA-hENT1 (green) and 3xFLAG-N48Q-hENT1 (red). Single transfected 3xFLAG-N48Q-hENT1 and wt 3xFLAG-ENT1 have a similar distribution to co-transfected cells (results not shown). Images represent a plane from a series of Z-stacks from one of three individual experiments. Scale bars represent 5 μm.

Transporters often require glycosylation for effective recruitment to the plasma membrane and thus function [13,15,16,32–40]. We therefore predicted that lack of glycosylation would interfere with trafficking of hENT1 at the plasma membrane and tested this using NBTI binding site saturation assays. These assays determine the number of total NBTI binding sites (where one NBTI binding site is equivalent to one hENT1 protein) present in a cell population. HEK293 cells transiently transfected with wt 3xFLAG-hENT1 showed a higher maximal NBTI binding (*B*_max_=41.5±2.9, *n*=3) compared with mock transfected cells (*B*_max_=0.441±0.027, *n*=3) (Figure 2A). N48Q mutant hENT1 transfected cells showed an NBTI binding (*B*_max_=13.5±0.45, *n*=3) which is greater than that seen in the mock transfection, but much lower (∼70%) than wt 3xFLAG-ENT1 suggesting that non-glycosylated hENT1 is greatly reduced in presence at the plasma membrane.

Similarly, when we assessed presence of wt hENT1 and N48Q hENT1 in HEK293 cells by microscopy, we noted that N48Q hENT1 has a primarily cytosolic distribution with some presence at the plasma membrane in punctate appearance (Figure 2B). In contrast, wt hENT1 protein is clearly present almost exclusively at the plasma membrane (Figure 2B). These findings are consistent when co-transfected (Figure 2B), or when observed separately as single transfections (results not shown).

### N48Q mutant ENT1 is non-functional in HEK293 cells

**Figure 3:**
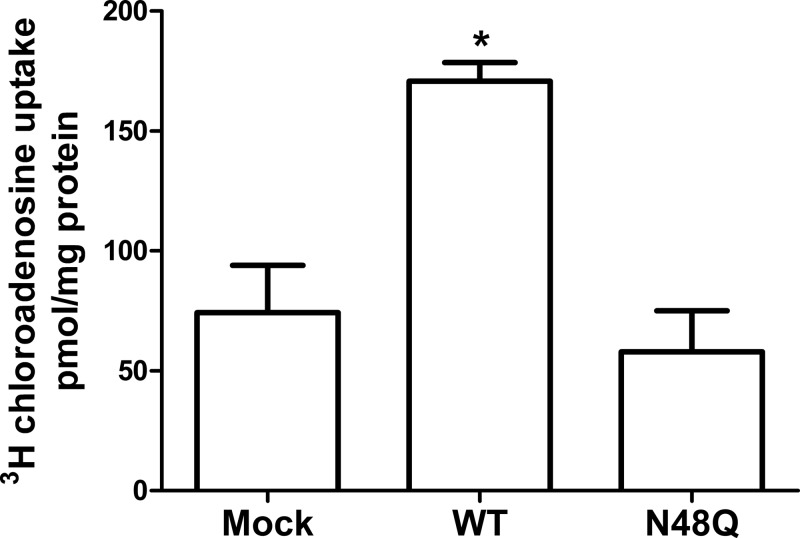
Glycosylation is required for sodium-independent nucleoside uptake in HEK293 cells HEK293 cells were either mock transfected, transiently transfected with wt 3xFLAG-hENT1, or with glycosylation mutant 3xFLAG-N48Q-hENT1. [^3^H]-chloroadenosine uptake was the same between mock transfected and N48Q-hENT1 transfected cells, but both were significantly less than wt hENT1 transfected cells. Graph represents pooled data from three individual experiments (*n*=3), with each condition conducted in sextuplicate. Error bars represent the mean ± S.D. (One-way ANOVA with Newman–Keuls multiple comparison post hoc test, ***P<0.0001).

N48Q hENT1, although less abundant, is still present at the membrane and therefore we predicted that we would see reduced, but not absent, transport. However, data show an almost complete lack of functional transport compared with wt hENT1. These data suggest that N48Q hENT1, even if present at the plasma membrane, is non-functional (Figure 3).

### ENT1–ENT1 co-immunoprecipitation is disrupted with mutation of glycosylation site

**Figure 4:**
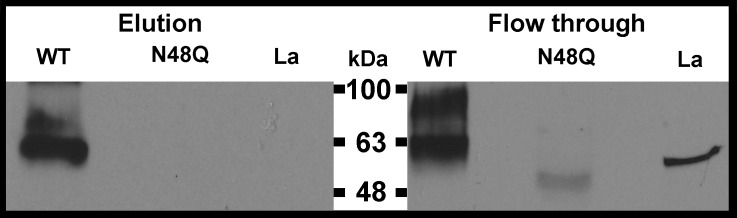
Co-immunoprecipitation analyses suggests glycosylation is required for hENT1–hENT1 interaction HEK293 cells were transiently transfected with HA-ENT1 as well as with the indicated construct (WT, wild type 3xFLAG-hENT1; N48Q, 3xFLAG-N48Q-hENT1; La, FLAG-hLa), lysed and co-immunoprecipitated using anti-HA beads (Thermo Scientific) as described in the Materials and Methods. Co-immunoprecipitation only occurred with wt 3xFLAG-ENT1 (100 μg lysate loaded to column), and not with N-glycosylation mutant 3xFLAG-N48Q-ENT1 (1000 μg lysate loaded to column) and negative control, cytosolic protein 3xFLAG-hLa (600 μg lysate loaded to column). Elution (bound protein) and flow through (unbound protein) were fractioned with SDS/12% (v/v) PAGE and immunoblotted with anti-FLAG antibody.

These data suggest that glycosylation of hENT1 may have a functional role in addition to assisting trafficking of the protein to the membrane. Oligomerization plays an important role for the proper function of other SLC members [41–43]. Our data show that ENT isoforms co-immunoprecipitate (ENT1–ENT1, ENT1–ENT2 and ENT2–ENT1) suggesting that ENTs form complexes with each other (N. Grañe-Boladeras, Z. Tarmakova, K. Stevanovic, D. Williams, L. Villani, P. Mehrabi, K.W.M. Siu, M. Pastor-Anglada and I.R. Coe, unpublished). ENT–ENT interactions or oligomerization may have important functional roles, which are yet to be identified (N. Grañe-Boladeras, Z. Tarmakova, K. Stevanovic, D. Williams, L. Villani, P. Mehrabi, K.W.M. Siu, M. Pastor-Anglada and I.R. Coe, unpublished). However, since we suspect that ENTs form dimers and we know that glycosylation of other transporters has been correlated with the formation of oligomers [20,21], we investigated the role of glycosylation of ENT1 in the formation of ENT dimers. As predicted, we observed that wt ENT1 co-immunoprecipitated with itself (HA-hENT1 with 3xFLAG-ENT1) but did not co-immunoprecipitate with 3xFLAG-N48Q-ENT1, or 3xFLAG-hLa (Figure 4) suggesting that N48Q mutant ENT1 is unable to form a complex with wt ENT1.

## DISCUSSION

Here, we provide evidence in support of the role of N-linked glycosylation in the function and localization of hENT1. We have confirmed previous reports [8–10] that the N-linked glycosylation site, N48, is unique for hENT1 expressed in a human cell line. The removal of this site significantly affects functionality of hENT1 which contrasts with previous data that suggested N48Q-ENT1 expressed in *S. cerevisiae* is functional [10]. This difference may be due to promiscuous glycosylation, which is known to occur in the yeast model [44–46] resulting in N-glycosylation at non-canonical sequences [47] which could play a compensatory role and restore function of N48Q-hENT1. Our study also suggests that glycosylation contributes to, but is not solely responsible for, correct ENT1 localization, since non-glycosylated ENT1 is present at the plasma membrane and that glycosylation is necessary for hENT1 function. This corroborates previous work which suggested that hENT1 mutant protein lacking the extracellular loop expressed in *X. laevis* had reduced hENT1 protein abundance at the plasma membrane [48]. Several members of the SLC family experience only a small or no functional effect when the N-linked glycosylation site is abrogated [11,12,49,50]. Typically, N-glycosylation leads to reduced transport activity as a consequence of reduced presence at the plasma membrane [13,15,16,32–40]. However, N-glycosylation may affect function in ways that are not related to trafficking or sorting. For instance, N-glycan deficient human erythrocyte anion transporter SLC4A1 (AE1) expressed in oocytes had reduced chloride transport yet had similar levels of surface protein abundance which authors attributed to non-ideal folding that effected function but not trafficking [51]. Aberrant glycosylation can also reduce protein half-life, which is a common characteristic of other over expressed N-glycosylation mutants from SLC family members [16,34,37,52]. Increased degradation of protein can result from changes in protein folding, as seen with OAT4 (SLC22A11) following N-glycan removal [53].

Our data suggest that glycosylation of hENT1 contributes to correct localization of the protein as well as functionality of the protein at the membrane and we propose that this may be correlated with glycosylation-dependent protein-interactions between hENT1 proteins at the membrane as proposed for other SLC members. Glycosylation of hOCT2 (SLC22A2) in HEK293 cells is required for the formation of hOCT2 dimers [42]. Similarly, serotonin (5-hydroxytryptamine) transporter (SLC6A4) monomer, when expressed in CHO hamster ovary cells, requires N-glycan addition to associate into functional homo-oligomers [41]. Our work (N. Grañe-Boladeras, Z. Tarmakova, K. Stevanovic, D. Williams, L. Villani, P. Mehrabi, K.W.M. Siu, M. Pastor-Anglada and I.R. Coe, unpublished) has revealed that ENT isoforms interact with each other (ENT1–ENT1, ENT1–ENT2, ENT2–ENT2, etc.) although the functional significance of this observation remains unclear. In the present study, we have shown that the loss of glycosylation abrogates interaction of ENT1 monomers and we therefore predict that oligomerization may be a fundamental form of regulation for the ENTs. This mechanism could explain previous data [54] where a large increase in hENT1 protein at the membrane yields a relatively small increase in the translocation of substrate, as well explaining the presence of distinct ENT1 populations (possibly differentially glycosylated variants) at the plasma membrane [55].

## CONCLUSION

We confirm that N48 is the single site of glycosylation of hENT1 in human cells. NBTI binding, immunofluorescence microscopy, chloroadenosine uptake assays and co-immunoprecipitation immunoblotting data suggest that hENT1 glycosylation at N48 is critical for the proper localization and function of the protein.

## Abbreviations

Asn: asparagine
HEK: human embryonic kidney
hENT1: human equilibrative nucleoside transporter 1
NBTI: *S*-(4-nitrobenzyl)-6-thioinosine
PNGase-F: peptide–*N*-glycosidase F
SLC: solute carrier
TMD: transmembrane domain
TTBS: tris-buffered saline and tween 20
wt: wild type
